# Automated data extraction from general practice records in an Australian setting: Trends in influenza-like illness in sentinel general practices and emergency departments

**DOI:** 10.1186/1471-2458-11-435

**Published:** 2011-06-06

**Authors:** Gösta TH Liljeqvist, Michael Staff, Michele Puech, Hans Blom, Siranda Torvaldsen

**Affiliations:** 1 NSW Public Health Officer Training Program, New South Wales Department of Health, Sydney, New South Wales, Australia; 2 School of Public Health and Community Medicine, University of New South Wales, Sydney, New South Wales, Australia; 3 Northern Sydney Central Coast Area Health Service, Hornsby, New South Wales, Australia; 4 Vale Medical Centre, Brookvale, New South Wales, Australia

## Abstract

**Background:**

Influenza intelligence in New South Wales (NSW), Australia is derived mainly from emergency department (ED) presentations and hospital and intensive care admissions, which represent only a portion of influenza-like illness (ILI) in the population. A substantial amount of the remaining data lies hidden in general practice (GP) records. Previous attempts in Australia to gather ILI data from GPs have given them extra work. We explored the possibility of applying automated data extraction from GP records in sentinel surveillance in an Australian setting.

The two research questions asked in designing the study were: Can syndromic ILI data be extracted automatically from routine GP data? How do ILI trends in sentinel general practice compare with ILI trends in EDs?

**Methods:**

We adapted a software program already capable of automated data extraction to identify records of patients with ILI in routine electronic GP records in two of the most commonly used commercial programs. This tool was applied in sentinel sites to gather retrospective data for May-October 2007-2009 and in real-time for the same interval in 2010. The data were compared with that provided by the Public Health Real-time Emergency Department Surveillance System (PHREDSS) and with ED data for the same periods.

**Results:**

The GP surveillance tool identified seasonal trends in ILI both retrospectively and in near real-time. The curve of seasonal ILI was more responsive and less volatile than that of PHREDSS on a local area level. The number of weekly ILI presentations ranged from 8 to 128 at GP sites and from 0 to 18 in EDs in non-pandemic years.

**Conclusion:**

Automated data extraction from routine GP records offers a means to gather data without introducing any additional work for the practitioner. Adding this method to current surveillance programs will enhance their ability to monitor ILI and to detect early warning signals of new ILI events.

## Background

Influenza poses a significant threat to public health, not only in the form of pandemics but also as seasonal epidemics. Each year, influenza causes 3-5 million cases of severe illness, and up to 500 000 deaths globally [1]. Surveillance of influenza-like illness (ILI) enables detection of the onset of influenza seasons and can provide early warning of new events. The National Health System in the United Kingdom has gained much from automated ILI data extraction in the QFlu program [2], and Brabazon *et al*. [3] obtained promising results with automated electronic data extraction from general practitioner (GP) records in Ireland.

In Australia, GPs play a key role in managing influenza and are often the first point of contact for patients with ILI [4]. Previous GP surveillance for ILI in Australia has required some form of additional work by GPs, either filling in reports or actively following surveillance-guided medical reporting using coded sections of medical records programs [5]. In NSW, notifications of laboratory-confirmed cases [6] and the Public Health Real-time Emergency Department Surveillance System (PHREDSS) [7] are the main data sources for influenza surveillance. While laboratory notification data include the outcomes of GP consultations, they provide notifications only of positive laboratory samples, severely limiting their value. Increasing numbers of GPs use software packages for keeping medical records [8], which has improved the uniformity and the quality of the records [9]. This may facilitate timely, comprehensive GP surveillance, which would enhance our ability to achieve early detection of new trends and identify and monitor clusters of cases [10].

We tested electronic automated data extraction of ILI data from routine GP records, assessed compatibility of the adapted software with GP office networks and investigated the timeliness of data and the sensitivity and specificity of automated ILI syndromic surveillance. We also compared local GP-reported ILI patterns with those seen at local emergency departments (EDs).

The aim of this study was to assess if syndromic ILI data can be extracted automatically from routine GP data by adapting an existing data extraction tool in Australia and to compare ILI data from general practice with ED data at a local level.

## Methods

### Ethical approval

Ethical approval for this study was provided by the Harbour Research Ethics Committee of Northern Sydney Central Coast Health.

#### Developing a new data extraction instrument

The Canning Division of General Practice in Western Australia has developed a software application capable of automated data extraction from GP records [11]. This tool is already in use for collection of data on chronic diseases, such as diabetes, but in its current configuration cannot be used for free-text searches. Discussions with local GP networks and potential participating GPs indicated that the Canning Tool was known and well accepted. Of 110 divisions of general practice in Australia, 85 use the Canning Data Extraction tool [11]. This information and the compatibility with GP medical records programs led us to select the Canning Data Extraction Tool for developing our new application.

On the platform of the existing Canning Tool, we developed a software application capable of automated data extraction from routine GP records. Free-text and coded information search combinations were used to identify cases that met an adapted case definition of ILI: *Any patient presenting with cough, fever or self-reported fever and fatigue *[12]. Free-text clinical information can be entered into the medical record, and provisional diagnoses can be recorded either in tick boxes, drop-down menus or free text. Information entered into tick boxes or drop-down menus is stored as codes rather than free text in the medical record database and requires a separate extraction process.

Building on the Canning Data Extraction Tool, we developed the Canning Flu Tool to conduct automated searches and extractions of both coded and free-text fields from two of the most commonly used GP medical record packages used in Australia (Best Practice and Medical Director 3). As the case definition has three distinct elements: respiratory symptoms, fever and fatigue, we programmed the tool to select words that described these elements (Table 1): a case was identified as ILI if at least one word in each of the three categories was present in the record. We also programmed the tool to recognise the expressions 'influenza', 'flu', 'ILI' and 'H1N1', and to use these as positive triggers when they appeared unaccompanied in the record. In order to address the possibility that any of these words was entered as a negative, i.e. no influenza or fever absent, each word was accompanied by a complex structured query language code for exclusion of negating terms.

**Table 1:** Canning Flu Tool search triggers

Canning Flu Tool trigger words for free text search		
**Case definition: A person presenting with cough, fever **≥ **38°C (or self-reported history of fever) and fatigue**		

The Canning Flu Tool identified as positive for ILI, any record that contained at least one word from each of the three columns (the search was not case sensitive)		

**Respiratory**	**Fever**	**Fatigue**

Cough	Chill	Fatigue
Dyspnoea	Fever	Lethargy
Respiratory Tract	Feverish	Lethargic
Infection	Febrile	Malaise
RTI	Pyrexia	Prostrate
Shortness of Breath	Pyrexic	Prostration
SOB	Rigor	Tired
Wheeze	Temp	Tiredness
Wheezing	Temps	Unwell
	Temperature ≥ 38	
	Temp ≥38	

The terms Flu, Influenza, ILI and H1N1 triggered a positive categorisation without the need for another word

Any description of influenza vaccination caused a negative categorisation.

#### Testing the Canning Flu Tool

The extraction program of the Canning Flu Tool was refined initially with a mock database and later with a real patient database. This allowed systematic testing of a variety of word combinations for both inclusion and exclusion in the ILI category. It also provided the opportunity to pilot-test the software in a real practice setting to ensure its compatibility with a practice computer network. This process was done with a group of participating GPs, who gave advice on common recording practices, and programmers to adjust and refine the data extraction process. The early version of the tool identified all visits for influenza vaccination as positive for ILI because the word "flu" appeared in these records. This resulted in a large number of false positives. The problem was corrected by adding specific exclusion criteria for the terms used to describe influenza vaccination. Similar processes were required to address the issue of patients visiting their GP to discuss pandemic influenza without actually having any symptoms.

After refinement, the tool's sensitivity and specificity for detecting cases that met the case definition were assessed by comparing its performance against the professional opinion of two public health physicians, who reviewed all the electronic records from two participating practices with Best Practice software during 1 week in August 2008.

### Program design and data collection

After refinement of the application, a study was conducted in the northern metropolitan area of Sydney, which has about 820 000 residents [13], five public EDs, and an estimated 1048 general practitioners in 328 practices [14], most being members of one of three local GP networks.

Eight GP sites participated in the study; in all, they saw up to 7000 patients per week (300 to 2000 patients per week per practice) during the surveillance period. Practices were selected on the basis of their geographical location in order to represent the study area and also to broadly match the catchment areas of the five public EDs in northern Sydney. Our local GP networks informed us that most GPs in the area use electronic records but that some do not use them to their full capacity, which would limit their use for surveillance purposes. We were guided by the GP networks in selecting practices, but this did not impose any limitations on selection of strategic sentinel practices. Our selection criteria were: use of either Best Practice or Medical Director 3, willingness to participate, and at least one practice within the catchment area of each ED.

We collected weekly reports from the beginning of May 2010 to the end of October 2010 in real-time and also collected retrospective data for comparison from all but one practice for the period May to October in 2007-2009 (the excluded practice did not open until 2009). The tool automatically extracted aggregated de-identified data and was installed either on a network server or on a work station in the practice. Data from all five local EDs were included, contributing a little over 2500 presentations per week. The average weekly counts of all ED presentations were 2749 (2007), 2777 (2008), 2972 (2009) and 2979 (2010). Weekly automated reports were delivered by the tool from each GP sentinel site including the total number of visitors per week and the count of ILI cases per week. Counts were conducted so that an individual could only contribute once to the number of patients who presented in any single week.

The weekly percentage of ILI cases in each practice was calculated automatically by the tool using the count of visits as denominator and the count of ILI cases as numerator.

When measuring sentinel activity, we used the weekly ILI percentage per site as provided in the weekly automated reports and calculated the arithmetic mean of these percentages to obtain a weekly measurement of ILI activity. We favoured this approach over weekly crude counts to ensure that summary measures were not unduly influenced by individual sites. It also made it possible to set a threshold for widespread influenza activity. We applied a 2% threshold in this study, which is consistent with practice elsewhere [15].

Feedback was provided to GPs by giving them access to a password-protected, secure website containing weekly surveillance reports, including both practice-specific and area-wide surveillance data and interpretations. Practice-specific information was coded in the reports so that it was identifiable only to the individual practice.

#### Comparing emergency department trends with general practice trends

We conducted a descriptive analysis to compare ILI trend curves. The data from both EDs and the sentinel GP sites were displayed in graphs using Microsoft Excel. GP data were temporally compared with data from the five local EDs collected by PHREDSS for the 2007-2009 periods and the 2010 period. PHREDSS extracts provisional diagnoses of ILI manually recorded in ED triage notes and coded according to ICD-9, ICD-10 or the Systematized Nomenclature of Medicine (SNOMED) for diseases caused by influenza [16]. At the time of this study, all the participating EDs used ICD-9.

In addition, we performed a free-text structured query language search of the ED records using the same search term as that used for GP records. The comparison of EDs and sentinel GP sites was performed retrospectively for May - October 2007, 2008 and 2009.

## Results

### Sensitivity and specificity of the Canning Flu Tool

Records from two practices for a 1-week period in August 2008 were reviewed (Table 2). The sensitivity of the tool was 96.3% and the specificity 99.7%. The positive predictive value was 76.5%, while the negative predictive value was 99.7%.

**Table 2:** Canning Flu Tool sensitivity and specificity table

**Sensitivity and specificity of the Canning Flu Tool**: Test against the clinical opinions of two Public Health Physicians using records from two practices (n 2518) over two weeks in August 2008			
**Gold Standard** (Public Health Physician's clinical opinion)			

**CFT**	**ILI present**	**ILI not present**	**Total**

**+**	26	8	34

-	1	2483	2484

**Total**	27	2491	2518

Legend. Canning Flu Tool (CFT), ILI (Influenza Like Illness)

Sensitivity 96.3%

Specificity 99.7%

Positive predictive value 76.5%

Negative predictive value 99.7%

### Sentinel GP data compared with PHREDSS data

**Figure 1:**
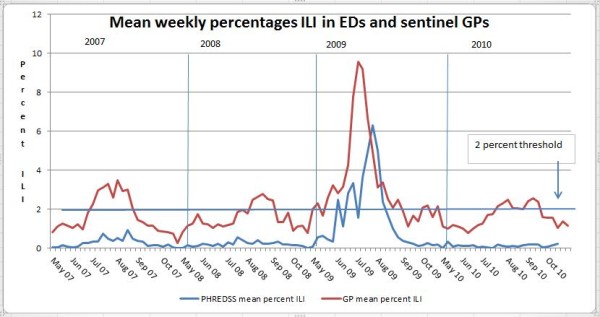
**Weekly mean percentages of ILI at GP sentinel sites and in five local EDs**. PHREDSS mean percentage ILI, GP mean percentage ILI.

Between 2007 and 2010, clear seasonal peaks can be seen in the GP data, the 2% threshold being exceeded in all four years (Figure 1). The percentage of ILI in the PHREDSS data remained below 1% in all non-pandemic years and did not provide a clear visual signal, while the sentinel GP sites showed clear seasonal peaks in each season monitored. In 2009, the GP peak preceded the ED peak by about 2 weeks; the amplitude was not great enough in the other years to allow a visual comparison.

In the non-pandemic ILI seasons monitored, the number of weekly ILI presentations in the five EDs ranged from 0 to 18 with percentages ranging from 0 to 0.93 in 2007 and from 1 to 13 with percentages from 0 to 0.54 in 2008, while the number in the GP sentinel sites ranged from 8 to 128 per week with percentages from 0.27 to 3.48 in 2007 and from 16 to 108 per week with percentages from 0.78 to 2.77 in 2008. Both the ED and the GP data showed clear increases associated with the pandemic season in 2009. The total count of visits per week in the five combined EDs ranged from 2441 to 3474 (the highest being in July 2009), with a mean of 2865. The highest total count of visits in the sentinel general practices was 7770 in October 2010. The total number of visits to GPs increased progressively from year to year, with the lowest count in June 2007 at 2432 (one of our sentinel practices opened in 2009).

### Free text extraction from ED data

**Figure 2:**
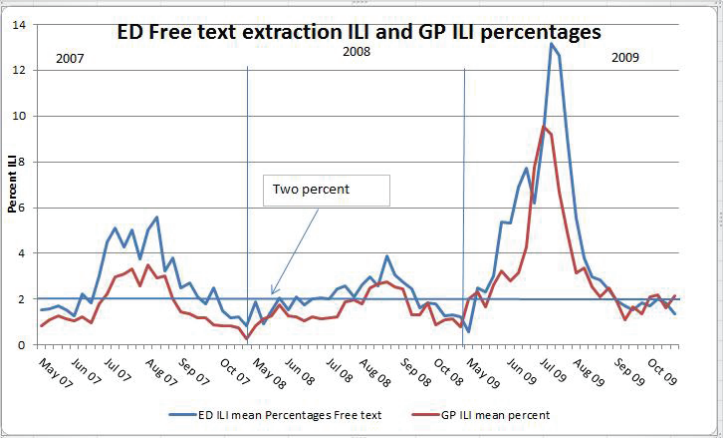
**Free-text search for ILI in EDs compared with GP sentinel sites (mean percentage)**. ILI weekly percentages in EDs and sentinel GPs with the same free-text extraction code used for both data sets.

A free-text search of ED records resulted in a seasonal curve that more closely matched the GP ILI curve than that provided by ED provisional diagnoses (as in PHREDSS). The percentage of syndromic ILI in EDs exceeded that at the GP sentinel sites when a free-text search was used (Figure 2).

## Discussion

Our results indicate that the Canning Flu Tool would be valuable for monitoring ILI activity in general practice and that it provides a sensitive signal of seasonal patterns of influenza like illness on a near real-time basis. Unlike other systems in Australia, the Canning Flu Tool can collect data from GP records without imposing any additional tasks. The tool produced a more robust signal than PHREDSS and is likely to detect increased ILI activity more reliably, chiefly because the counts are higher and less volatile, while cases of ILI as per the case definition are still detected with high sensitivity and specificity. The sensitivity of PHREDSS was enhanced by application of a free-text search, which detected more cases than provisional diagnostic coding and resulted in a temporal pattern very similar to that from GP data.

Adding the Canning Flu tool to NSW and Australian surveillance practice could improve the detection of seasonal onset of influenza like illness and hence assist GPs to prepare for the inevitable influenza season each year, and allow timely community messages and increased infection control measures, which are vital parts of both pandemic and seasonal preparedness at frontline services such as GPs and EDs [17]. Furthermore, this use of electronic data opens up a previously largely unmonitored portion of ILI activity and hence provides valuable epidemiological knowledge, resulting in increased ability for early detection of new trends. This will further inform and enhance public health responses.

By design, the Canning Flu Tool identifies ILI rather than influenza specifically. It provides syndromic information and seasonal trend observations and should hence be used in combination with and as a complement to laboratory testing and other surveillance methods. Furthermore, the study was conducted in a metropolitan area with relatively high socioeconomic status, and we cannot assume that the findings are applicable to the whole population of NSW or Australia.

One of the two medical record software packages accessed by the Canning Flu Tool allowed free-text searching of the progress notes field, while the other package encrypts the progress notes field. The sensitivity of the tool might be lower in practices in which the latter software package is used, and this should be considered when recruiting GPs to a surveillance system based on electronic medical records.

For further analysis of trends, a formal time series analysis would be ideal, but this has some pitfalls, as described by Zheng et al. [18], and requires rich data, i.e. daily counts, which was beyond the scope of this study.

The Canning Flu Tool can provide timely, locally relevant information for GPs to enhance infection control measures within their practices, give targeted messages to their patients and have greater collaboration and communication with public health authorities. Future studies are recommended to develop additional syndromic applications for automated data extraction from GP records. The Canning Flu Tool could be expanded to other relevant syndromes, either for locally specific programs or for state- or nationwide surveillance. This might result in a significant improvement in surveillance not only of infectious diseases but potentially also of chronic conditions.

As electronic records are being used by increasing numbers of GPs in Australia, and automated data extraction from electronic records has proven to be useful in the United Kingdom and Ireland [2,3], we consider that this could become an efficient surveillance method in the Australian setting, where general practices are mostly private.

## Conclusion

This study demonstrates that the Canning Flu Tool performs well and has the potential to be useful within the Australian general practice setting. Automated data extraction from routine GP records offers a means to gather data without introducing any additional work for the practitioner. Adding this method to current surveillance programs will enhance their ability to monitor ILI and to detect early warning signals of new ILI events.

## Competing interests

None. The Canning Division of General Practice was consulted and remunerated for developing their tool to the specifications of the study.

The chief project manager was employed by the NSW Department of Health with an affiliation to the University of New South Wales.

## Authors' contributions

GL was the lead author and project manager; he collected and analysed data and provided interpretation. MS provided study design support and guidance as well as data analysis support, assessment of the Canning Flu Tool (sensitivity and specificity, data analysis) and editorial support. MP participated in assessment of the Canning Flu Tool (sensitivity and specificity, data analysis) and gave editorial support. HB Tested the Canning Flu Tool in a practice setting, provided input on GP recording practices for the development of search triggers and gave editorial support. ST contributed to the analysis and interpretation of data, critically revised the manuscript for intellectual content and gave editorial advice. All the authors approved the final version of the manuscript.

## Pre-publication history

The pre-publication history for this paper can be accessed here:

http://www.biomedcentral.com/1471-2458/11/435/prepub
